# Immobilization of different biomolecules by atomic force microscopy

**DOI:** 10.1186/1477-3155-8-10

**Published:** 2010-05-17

**Authors:** Michael Breitenstein, Ralph Hölzel, Frank F Bier

**Affiliations:** 1 Fraunhofer Institute for Biomedical Engineering, Department of Nanobiotechnology and Nanomedicine, Am Mühlenberg 13, 14476 Potsdam, Germany; 2 University of Potsdam, Institute for Biochemistry and Biology, Karl-Liebknecht-Str. 24-25, 14476 Potsdam, Germany

## Abstract

**Background:**

Micrometer resolution placement and immobilization of probe molecules is an important step in the preparation of biochips and a wide range of lab-on-chip systems. Most known methods for such a deposition of several different substances are costly and only suitable for a limited number of probes. In this article we present a flexible procedure for simultaneous spatially controlled immobilization of functional biomolecules by molecular ink lithography.

**Results:**

For the bottom-up fabrication of surface bound nanostructures a universal method is presented that allows the immobilization of different types of biomolecules with micrometer resolution. A supporting surface is biotinylated and streptavidin molecules are deposited with an AFM (atomic force microscope) tip at distinct positions. Subsequent incubation with a biotinylated molecule species leads to binding only at these positions. After washing streptavidin is deposited a second time with the same AFM tip and then a second biotinylated molecule species is coupled by incubation. This procedure can be repeated several times. Here we show how to immobilize different types of biomolecules in an arbitrary arrangement whereas most common methods can deposit only one type of molecules. The presented method works on transparent as well as on opaque substrates. The spatial resolution is better than 400 nm and is limited only by the AFM's positional accuracy after repeated z-cycles since all steps are performed in situ without moving the supporting surface. The principle is demonstrated by hybridization to different immobilized DNA oligomers and was validated by fluorescence microscopy.

**Conclusions:**

The immobilization of different types of biomolecules in high-density microarrays is a challenging task for biotechnology. The method presented here not only allows for the deposition of DNA at submicrometer resolution but also for proteins and other molecules of biological relevance that can be coupled to biotin.

## Background

Bottom-up fabrication of defined nanostructures on solid surfaces requires immobilization of different addressable biomolecules as anchors. Nanometer scaled deposition of minute sample volumes was first introduced by Mirkin and co-workers [1,2] and was realized by dip-pen nanolithography [3-5] where the tip of an atomic force microscope (AFM) is used to deposit reactive compounds directly on a surface.

However, it is difficult to use such lithographic methods for a fast and easy deposition of different biological compounds on the same carrier. So far no techniques were reported capable of deposing more than two kinds of biomolecules with a single tip. In addition, these biomolecules have to match the needs of the used spotting method. In the group of Klenerman [6,7] a method for the controlled deposition of biomolecules using a scanning nanopipette was developed. They deposited biomolecules by electrophoretic flow applying a local voltage between the nanopipette and the surrounding medium that covers the functionalized surface. An alternative method with high spatial resolution is based on synthesis on the chip: Fodor and co-workers [8] developed a method to produce microarrays by repetitively uncovering photo-labile protecting groups on an activated silicon wafer with UV irradiation through a mask. Deprotection of the photolabile groups leads to coupling of the modified compounds. This method was commercialized by Affymetrix, who developed microarray feature sizes of 10 × 10 μm^2 ^but is limited to oligonucleotides and peptides. However their length is restricted due to incomplete reactions [9]. For higher quality and more versatility of biomolecule species, the deposition of presynthesized molecules is advantageous. Here we present a novel, universal technique that allows immobilizing different types of biomolecules in a well defined, high resolution pattern by using only one single AFM-tip. Chemical and fluidic properties of the molecules that have to be immobilized by this method may differ and do not have to match, as it is the case with other methods. This approach is a first step towards generating individually patterned nanostructures fixed on surfaces and allows the construction of DNA anchored structures.

The method presented is a microcontact printing, still it is highly flexible and pattern independent. The protocols and methods that are established in microcontact printing, e.g. [10], have recently been shown to be applicable to the preparation of microscopically small features [11] and, hence, could be combined with our approach for immobilizing several different biomolecules.

This work is aimed at the development of a universal method for the production of micrometer scaled arrays with several different immobilized biomolecules. For scaling down feature size and for greater flexibility an atomic force microscope (AFM) was used. The AFM-tip is utilized like a pen and allows small volume deposition of reactive compounds. A crucial prerequisite for the immobilization of different biomolecules is to avoid any cross contaminations. This can be achieved by either cleaning or replacing the tip. However, replacing would result in a decreased accuracy due to long distance-moving of the tip, whereas cleaning might precipitate cross contaminations and, furthermore, would result in decreased accuracy because of the additional movement. To solve this problem, neutravidin is used as a natural, highly reactive linker for biotinylated biomolecules and is deposited by a single AFM-tip. This results in two significant advantages: Besides an improved accuracy of spot positions, optimization and adaptation steps for deposition are reduced to a minimum, since only one chemical compound, neutravidin, is to be deposited.

## Results and Discussion

**Figure 1:**
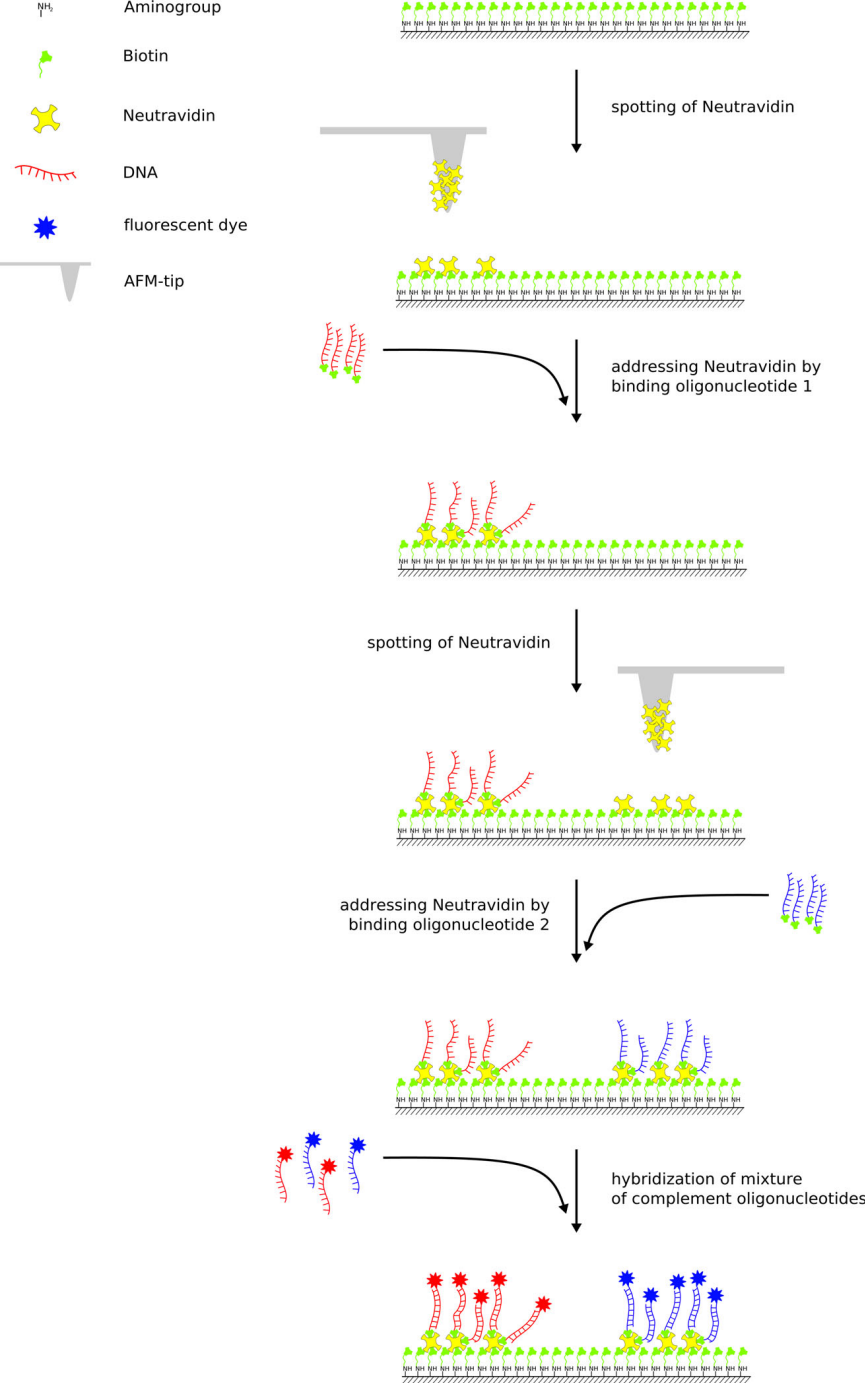
**Illustration of the spotting process**. The first spot is generated by addressing the spotted neutravidin with biotinylated oligonucleotides. All following spots can be created by repeated spotting and addressing without replacing the surface. Here, the whole cycle is completed after two passes. By hybridization with complementary ssDNA which is fluorescently labeled the array can be visualized.

We employ common AFM-tips without further modification. They are well suited to apply glycerol based ink containing neutravidin on a homogeneously biotinylated glass surface with high precision. Because of the transparent and non-conducting glass, the array is accessible for further analysis, e.g. optical microscopy or conductance measurements. Neutravidin is used because it offers specific binding sites for biotin. It captures up to four biotin molecules and forms one of the strongest non-covalent bonds with an unbinding force of up to 250 pN [12] giving high stability. The generated spots, where the neutravidin is bound to the covalently immobilized biotin, remain reactive for at least one further biotin molecule due to its four binding sites. Here it is used as a natural linker where biotinylated biomolecules can couple. Biotinylation is commonly used and commercially available for most kinds of biomolecules. Therefore, DNA, proteins, nano-beads and many other molecules such as dyes can be immobilized. The technique is performed sequentially and a further spotting step with neutravidin follows when the neutravidin array has been incubated with the first biotinylated molecule species. The procedure is concluded by a further incubation with the next type of biotinylated biomolecule. This cycle of spotting and biomolecule binding can be repeated several times. In Figure 1 the whole procedure is illustrated being completed by complementary DNA hybridization. Time consuming incubation and coupling steps can be avoided due to the fast binding process. The whole spotting can be carried out under ambient temperature and humidity conditions reducing executional complexity.

**Figure 2:**
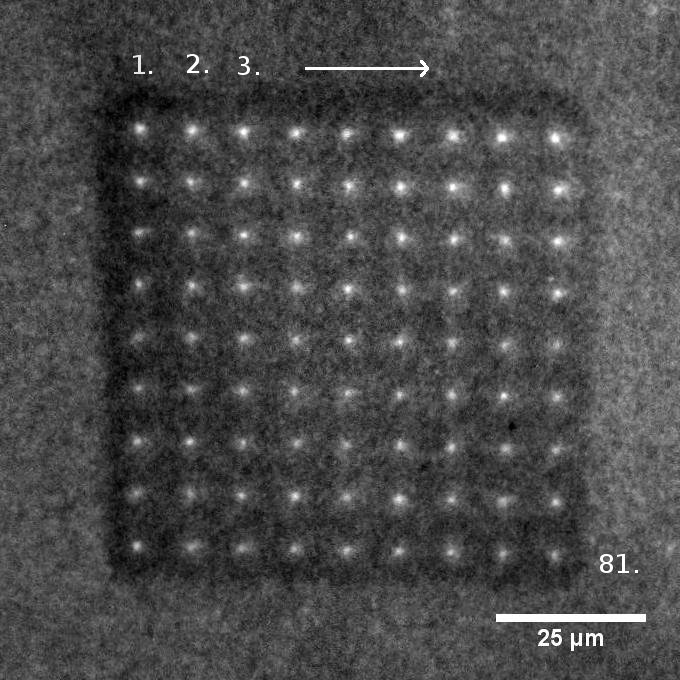
**Fluorescence micrograph**. Spotted 9 × 9 array on a biotinylated glass surface. After spotting neutravidin, the array was incubated with the biotinylated dye DY-547 and finally washed with water. The distance between each spot centre is 9 μm. The systematic reduction of spot size is owed to depletion of the tip-loading.

To test the procedure it was applied for the preparation of an array of biotinylated dyes. First a glass slide was gas-phase silanized with aminopropyltriethoxysilane (APTES) to provide the surface with reactive aminogroups that can couple with NHS-biotin for the following homogeneous biotinylation. The biotinylation was carried out over night in phosphate buffer. Then the slides were washed with water and dried with nitrogen. Like this, they can be stored at room temperature for several weeks. In addition to neutravidin and water, the ink contained glycerol to delay evaporation. Spotting started with loading the AFM-tip with ink, utilizing a micro capillary. The AFM-tip was immersed into the droplet that formed at the end of the capillary. A micromanipulator helped to move the ink reservoir. Once the tip was loaded with ink, the script-based spotting protocol was started by approaching and contacting the surface in succession. For each spot the neutravidin loaded tip remained in contact with the surface for four seconds. Due to the fast binding between neutravidin and surface-bound biotin, a five minutes incubation time after spotting the complete array was sufficient. Accordingly, the target substance that had to be immobilized - here demonstrated by a biotinylated fluorescent dye DY-547 in carbonate buffer - was also incubated for five minutes to bind to the spotted neutravidin. Incubation was performed under the same environmental conditions as spotting. Figure 2 presents a fluorescence micrograph of an AFM-spotted 9 × 9-array on a glass slide. The grid measures 9 μm between spot centers with 1.5 μm diameter.

**Figure 3:**
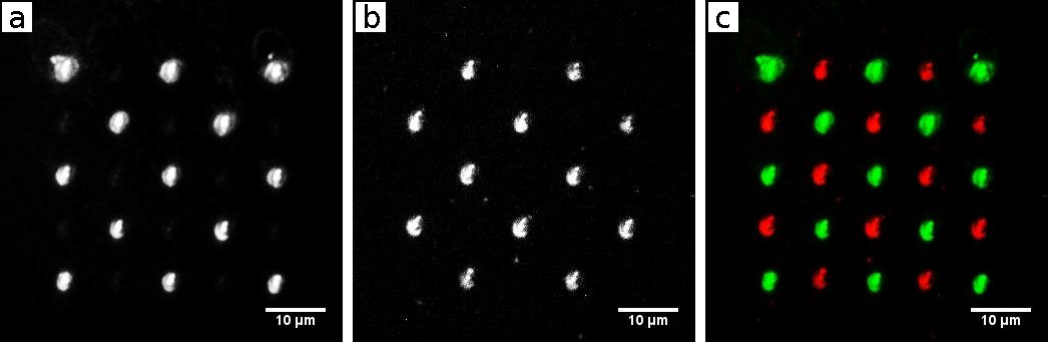
**Two component array**. Fluorescence photomicrograph of a sequentially spotted, two component array. a) DY-547-channel, b) DY-647-channel, c) merged image of a) and b) with each dye color coded differently.

Immobilization of more than one substance is achieved by dividing the array into two sub-arrays which are generated sequentially. The first neutravidin array was spotted, followed by immediate incubation of the biotinylated fluorescent dye DY-547. Washing of unbound dye was performed with PBS and subsequently ultrapure water without physically moving the specimen. After that the second neutravidin array was generated exactly within the same region to complete the final array. This was achieved without replacing or moving the surface where we spotted on so that an alignment was unnecessary. At this point the array comprised of a fluorescent dye coupled to neutravidin and new, unaddressed, neutravidin. The latter then were addressed by the second biotinylated dye through incubation with DY-647-Biotin. After washing again with PBS and ultrapure water the array was visualized by fluorescence microscopy. The successively immobilized dyes were excited separately: Figure 3a shows DY-547-Biotin addressed spots (excited with 545 nm), Figure 3b shows DY-647-Biotin addressed spots (excited with 620 nm). The merged image of the array is shown in Figure 3c.

**Figure 4:**
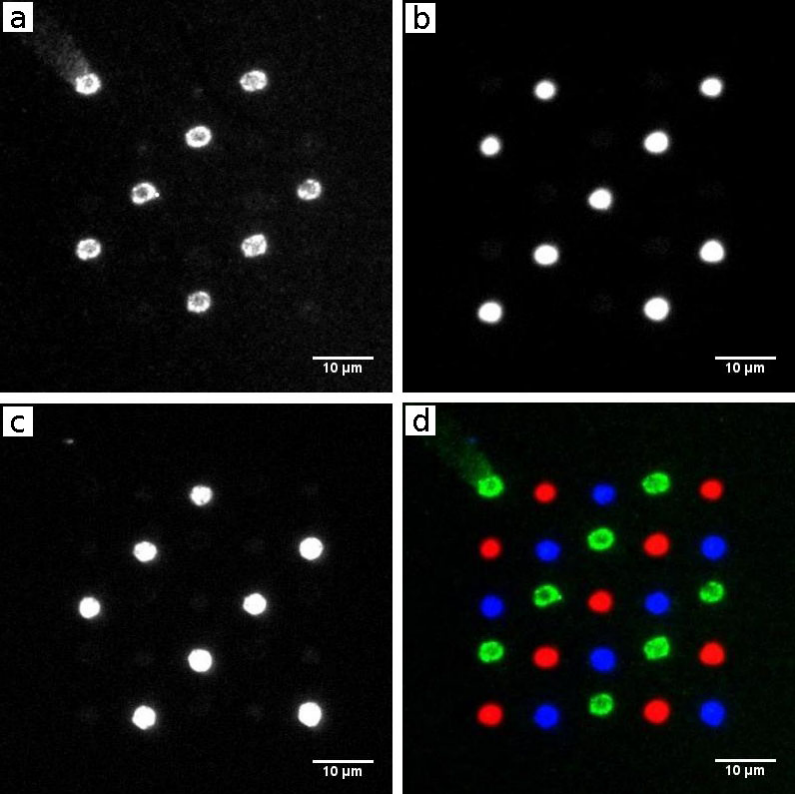
**Three component array**. Fluorescence micrograph of three hybridized Cy3, Cy5 and Atto495 functionalized oligonucleotides after sequential spotting of neutravidin. The three complementary oligonucleotides are represented by red, green and blue spots, respectively. a) Cy3-channel, b) Cy5-channel, c) Atto-495-channel, d) merged image.

**Figure 5:**
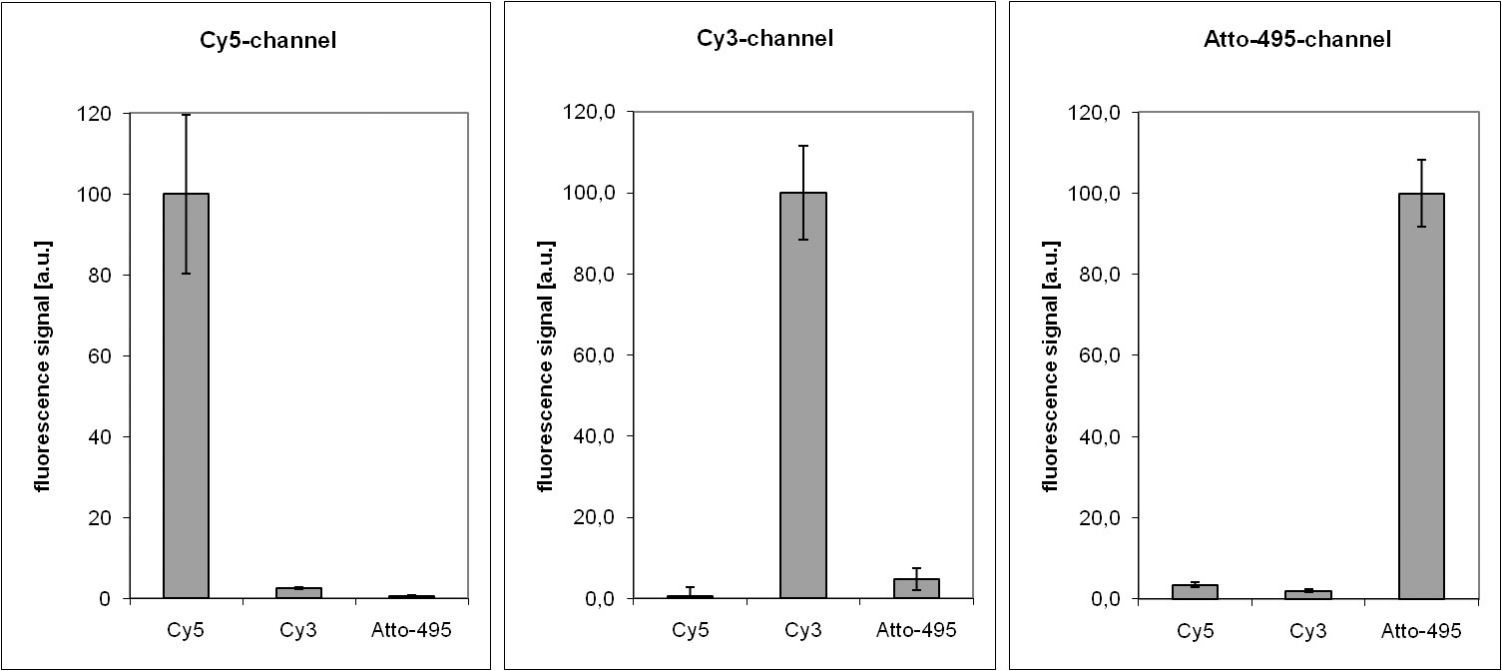
**Cross contamination**. Measured fluorescence signals of individual spots in the Cy3, Cy5 and Atto-495-channel.

For the construction of defined structures on the nanometer scale nucleic acids are very promising compounds. Their ability to form structures on a molecular level by self-assembly [13,14] is the basis for new technical applications. Immobilization of DNA-molecules was tested as follows. To prevent unspecific binding of DNA, the targeted surface was blocked. Strategies using biological (e.g. BSA from AppliChem GmbH, 64291 Dortmund, Germany) and synthetic (e.g. Roti-Block from Carl Roth GmbH & Co. KG, Karlsruhe, Germany) blocking methods were tested as well as casein (Sigma Chemical CO, MO 63178 USA) blocking which turned out to work best (data not shown). In respect to cross-hybridization and hybridization efficiency Niemeyer and colleagues [15,16] optimized several 22 base pair sequences. Three of their published DNA sequences were chosen. The single stranded 5'-biotinylated forms were immobilized in the same way as described above. In the first immobilization step the biotinylated strand (RcF6) was incubated, followed by the second (LcF5) and finally the third one (RcF2) to generate a three component array. After washing residual DNA, the array was ready for hybridizing with a mixture of three oligonucleotides being complementary to each of the DNA sequences and having been labeled by three different fluorescent dyes. According to the self-assembling capability of DNA the labeled DNA strands hybridized to the immobilized oligonucleotides (see Figure 4). It is evident that the first incubation led to a complete occupation of all neutravidin spots. Cross talk between the three channels of the array is shown in Figure 5: Cy5-channel: 2.5% for Cy3 and 0.6% for Atto-495; Cy3-channel: 0.5% for Cy5 and 4.8% for Atto-495; Atto-495-channel: 3.5% for Cy5 and 2.0% for Cy3. To compare the resulting signals with respect to cross talk between the filter-cube combinations, fluorescence signals of individual one-substance arrays without any cross contamination were tested. The resulting signals were found to be in the same range as those measured in the array in Figure 4 (data not shown). Consequently cross contaminations are negligible with this method.

## Conclusions

A novel approach has been presented for the high resolution immobilization of multiple biomolecules on a solid glass support and within the same array. The unique feature of the method is that optimization of the spotting protocol can be reduced to just one spotting substance, regardless of what kind of biotinylated mixture of biomolecules is to be arranged. Two completely different species of molecules have been used to demonstrate the sequence of working steps: biotinylated fluorescence dyes and single stranded DNA. In addition to the flexibility of the whole process, the possibility to treat the surface prior to each spotting step simplifies the whole procedure considerably. The unaffected biochemical activity of the immobilized molecules was shown by hybridization of a mixture of fluorescently labeled complementary oligonucleotides. In general, the results pave the way for creating surface bound and well addressed nanostructures that are based on functional biomolecules.

In subsequent steps the spots may be used to immobilize a variety of arbitary molecular species in a single array. In contrast to gold-thiol chemistry, the glass remains accessible for all types of optical microscopy. The whole immobilization procedure can be performed on transparent as well as opaque surfaces. The solid glass support can be employed for many applications where a non-conductive, transparent surface is needed. The procedure is also interesting for other structuring methods like micro-contact printing. We expect that existing nanolithography methods can be upgraded using the presented method.

All experiments have been carried out with nanolithographic tools, such as standard AFM-tips and script-based execution. It would also be possible to modify this general procedure for microcontact printing methods to fulfill the needs of industrial mass production. Further investigations will aim to reduce feature size to the sub micrometer range to enable fabrication of DNA-based nano-structures.

## Methods

### Silanization and biotinylation

Glass slides (Menzel Gläser, Menzel GmbH & Co. KG, 38116 Braunschweig, Germany) were cleaned with ultrasound in acetone for 15 minutes and again in ethanol (acetone and ethanol were obtained from Carl Roth GmbH & Co. KG, Karlsruhe, Germany). After rinsing with ultrapure water, the slides were put into NaOH (10 M) for 1 minute and washed thoroughly with water. Drying was carried out in a centrifuge (Varifuge 3.0R, Heraeus) for 1 minute at 2 g. In vapor phase at 120°C the silanization with 3-Aminopropyltriethoxysilane (Fluka Chemie GmbH, 89552 Steinheim, Germany) was executed in a sealed beaker and finished after 60 minutes. Silanization was tested by contact angle measurements by the sessile-drop tangent method - contact angle system from Dataphysics OCA30. For biotinylation, Sulfo-NHS-Biotin (20 mg) (Thermo Scientific, IL 61101 USA) was dissolved in DMSO (1 mL) (Carl Roth GmbH & Co. KG) because of its low stability and moisture-sensitivity. The DMSO solved Sulfo-NHS-Biotin can be stored at -20°C with desiccant. Sulfo-NHS-Biotin (10 mL) solution was added to Na_2_HPO_4 _(100 mM, 21 mL), NaCl (150 mM) buffer at pH 7.4. Incubation of 5 silanized glass slides took place for 3 hours at room temperature. Slides were washed with PBS and rinsed with water. Blocking was carried out by incubating the glass slides in a freshly preparated, 0.1% (w/v) solution of blocking reagent CA from Applichem in 100 mM Tris-Cl. For cleaning, slides were washed three times for 5 minutes in Tris-Cl and finally rinsed with ultrapure water. NaOH, Na_2_HPO_4_, NaCl, PBS and blocking reagent CA were obtained from AppliChem GmbH, 64291 Dortmund, Germany.

### Array preparation

Addressing the spotted neutravidin (Thermo Scientific, IL 61101 USA) was accomplished by incubation of the biotinylated substance that had to be immobilized. Biotinylated dyes (Dyomics GmbH, 67745 Jena, Germany) as well as the biotinylated oligonucleotides (Biomers.net GmbH, 89077 Ulm, Germany) were diluted in carbonate buffer pH 9.0 to a final concentration of 0.5 mM. Incubation time for binding was 5 minutes and was stopped by washing with 1× PBS-buffer and ultrapure water. Oligonucleotides were treated with additional hybridization of fluorescently marked ssDNA. Hybridization was carried out in the dark for 30 minutes at 37°C and 80% relative humidity. Sequences of the oligonucleotides (Niemeyer *et al.*): LcF5: 5'-cttatcgctttatgaccggacc-3' (5': Biotin); RcF6: 5'-caatgaaacactaggcgaggac-3' (5': Biotin) and RcF2: 5'-gtcggttctaagaaaatggcgg-3' (5': Biotin). The three fragments were diluted in TE-buffer (50 mM Tris-Cl, 100 mM NaCl, AppliChem GmbH) to a final concentration of 1 mM. Washing was carried out with PBS-buffer and stringent washing with ultrapure water.

### Spotting

The atomic force microscope CP-II from Veeco (Santa Barbara CA, 93117 USA) and AFM-tips from NanoSensors (NanoAndMore GmbH, 35578 Wetzlar, Germany): DT-CONTR (force constant: 0.2 N/m; resonance frequency: 13 kHz) was used. Movement of the AFM-tip and execution were controlled by the diNanolithography Software V.1.8. Approaching the biotinylated glass slide was achieved in contact mode with 3.4 mN contact force. The tip remained in contact for 4 seconds and changed to the next spotting positions by retraction. Ink was supplied by a hypodermic needle of Popper & Sons, Inc. (N.Y. 11040 USA).

### Microscopy

Fluorescence microscopy was carried out with an upright epifluorescence microscope Olympus A BX51 (objective: UPlanFL N; 40×/0.75). Fluorescence detection was accomplished with the following filter-cube combinations: Cy3 and DY-547 detection: excitation filter (Ex) BP 545/25, dichromatic mirror (Dm) 565, emission filter (Em) LP 605/70; for Cy5 and DY-647 detection: Ex BP 620/60, Dm 660, Em BP 700/75; and for Atto495 detection: Ex BP 460 - 495, Dm 505, Em LP 510 - 550. For illumination a mercury arc lamp (100 W, OSRAM GmbH, 81543 München, Germany) was used. Image acquisition was carried out with a CCD camera from Finger Lakes Instrumentation (FLI, New York 14485 USA; CM10-ZME; 6.8 μm pixel pitch; 2184 × 1472 pixels) in combination with FLIGrab Software V1.10. Image editing was realized with ImageJ V1.42q.

## Competing interests

The authors declare that they have no competing interests.

## Authors' contributions

MB performed the experiments and designed most of them. RH and FB conceived of the study and participated in its design and coordination. The authors participated in the evaluation and interpretation of the experiments. MB prepared the first draft of the manuscript and all authors contributed to its finalization.
